# Psychological wellbeing of Italian students and clinical assessment tools at university counseling services during the COVID-19 pandemic: a systematic review

**DOI:** 10.3389/fpsyg.2024.1388419

**Published:** 2024-08-13

**Authors:** Giovanna Celia, Gianluigi Serio, Eugenio Trotta, Francesca Tessitore, Mauro Cozzolino

**Affiliations:** ^1^Department of Humanities, Literature and Cultural Heritage, University of Foggia, Foggia, Italy; ^2^Department of Humanities, Philosophy and Education, University of Salerno, Salerno, Italy

**Keywords:** psychological wellbeing, Italian university students, COVID-19 pandemic, assessment, psychological counseling, systematic review

## Abstract

**Introduction:**

The COVID-19 pandemic brought about unprecedented problems for contemporary society. Among the numerous challenges faced following the spread of the virus, the need to provide assessment tools and remote psychological assistance certainly deserves particular attention. Indeed, this profound paradigm shift in psychological assessment and care occurred during a period of fragility for people already suffering from the restrictions imposed to stem the spread of the virus. One population particularly affected by the pandemic was students, including university students. The latter faced the pandemic in a particularly critical formative period.

**Methods:**

For these reasons, our systematic review has two main objectives: (i) identify the assessment tools and psychological dimensions most used/investigated during the pandemic in Italian university students; (ii) systematize and deepen our knowledge about the impact of the pandemic on the psychological wellbeing of Italian university students. Our search used PRISMA 2020 guidelines on Web of Science, Pubmed, Scopus, and EBSCOHost.

**Results:**

The results indicated that the psychological dimensions most investigated in university students during the pandemic were anxiety and depression. At the same time, the most used assessment instruments were the State-Trait Anxiety Inventory (STAI-Y) and the Beck Depression Inventory-II (BDI-II). Furthermore, it is clear from the results that the pandemic has harmed the psychological wellbeing of university students. Finally, we dedicated a section to discuss the interventions implemented by university counseling services during the pandemic.

**Discussion:**

This review could improve the work of university counseling services in this post-pandemic period and contribute to developing specific screening and assessment programs for future emergencies.

## 1 Introduction

The 2019 coronavirus disease (COVID-19) pandemic rapidly spread globally, leading the World Health Organization (WHO) to declare a state of global pandemic in March 2020 (WHO, 2020). Despite the strategies implemented to contain the virus, which have significantly slowed its spread and had a substantial impact on reducing transmission (Flaxman et al., 2020; Hsiang et al., 2020), the pandemic has profoundly affected various aspects globally, including mental health. Worldwide scientific literature illuminates the pervasive impact of COVID-19 on mental health, underscoring its profound repercussions within the general population. In particular, current literature suggests that both contracting the COVID-19 virus and the measures adopted for containment have contributed to the increased incidence and development of depression, anxiety disorders, stress, panic attacks, somatization disorders, sleep disturbances, symptoms of post-traumatic stress, manifestations of suicidal behavior, and social isolation (Hossain et al., 2020; Hwang et al., 2020).

Among the demographic cohorts most profoundly affected by the pandemic, young adults and university students have emerged as particularly vulnerable populations (Kross et al., 2013), with higher rates in women and younger students (Almeida et al., 2020; Qiu et al., 2020). The disruption of daily routines, the erosion of social networks, and the uncertainty surrounding academic pursuits have compounded the psychological burden borne by this demographic cohort. Compared to the general population, a higher prevalence of mental health issues in university students has been evidenced (Li et al., 2021; Villani et al., 2021). Various studies have shown, in fact, a significant rise in stress, anxiety, depression levels (Alkhamees et al., 2020; Cao et al., 2020; Savage et al., 2020; Wang et al., 2020; Wathelet et al., 2020; Chen and Lucock, 2022; Faisal et al., 2022), and, more broadly, a deterioration in mental health among university students (Browning et al., 2021; Villani et al., 2021; Kim et al., 2022). Regarding the different type of university courses, research has mainly focused on medical students whose mental health status has already been demonstrated to be poorer than that of general population especially due to academic stress (Thuma et al., 2020). A recent systematic review (Jia et al., 2022) which compared studies investigating the rates of mental health in medical students compared to general population, showed that the prevalence of depression and anxiety among medical students during COVID-19 was relatively higher than those of the general population and the healthcare workers. However, evidence produced contradictory results on this point. For example, comparing medical students with non-medical students (such as economics, law, education, and history), some studies showed that medical and healthcare students reported lower prevalence of moderate to severe levels of depression, anxiety, and stress symptoms (Sundarasen et al., 2020; Leroy et al., 2021; Xiong et al., 2021). Regarding depression levels, students who engaged in health-science-related studies had less risk of developing depression compared with students in other fields of studies (Mekonen et al., 2021).

Recognizing the gravity of the situation and the possible negative repercussion of pandemic on university students' wellbeing, universities worldwide have implemented various psychological interventions and counseling initiatives aimed at mitigating the adverse effects of the pandemic on student mental health. These interventions encompassed a spectrum of approaches, ranging from individual counseling sessions and support groups to online mental health resources and virtual wellness workshops (Son et al., 2020). In general, several studies have shown the effectiveness of digital mental health interventions in improving depression, anxiety, and overall psychological wellbeing among university students (Musiat et al., 2014; Saleh et al., 2018; Lattie et al., 2019; Viskovich and Pakenham, 2020). During pandemic, the effectiveness of online interventions in promoting the mental health and wellbeing of university students has been demonstrated (Riboldi et al., 2023).

Within the Italian context, the impact of COVID-19 on mental health has been a subject of growing concern and scholarly inquiry. Research by Giallonardo et al. (2020) and Rossi et al. (2020) well elucidated the disproportionate burden of mental health disorders among segments of the Italian population, shedding light on the intersecting socio-economic and psychosocial factors shaping vulnerability and resilience in the face of adversity.

Despite the burgeoning body of research on the broader impact of COVID-19 on mental health in Italy, there remains a conspicuous gap in our understanding of its specific ramifications for university students. Moreover, as the pandemic unfolded, Italian universities endeavored to support students grappling with the psychological fallout of the crisis, instituting online psychological interventions directed to university students. Yet, the efficacy and accessibility of university counseling services and mental health interventions during pandemic remain underexplored terrain, necessitating rigorous empirical inquiry to inform evidence-based practice and policy.

### 1.1 Aims of the study

Our systematic review has two main objectives: (i) identify the assessment tools and psychological dimensions most used/investigated during the pandemic in Italian university students; (ii) systematize and deepen our knowledge about the impact of the pandemic on the psychological wellbeing of Italian university students. In addition, we dedicate a section to discussing the interventions implemented by university counseling services during the pandemic.

## 2 Method

### 2.1 Information sources, search strategy, and eligibility criteria

This review was conducted according to the Preferred Reporting Items for Systematic Reviews and Meta-Analyses (PRISMA) guidelines (Page et al., 2021). On April 14^th^, 2023, four electronic databases were consulted: Pubmed, Web of Science, Scopus, and EBSCOhost (APA PsycInfo). We limited our selection to scientific studies published in English and Italian languages from March 2020 to April 2023. We used words such as “Assessment,” “University,” “School,” “Psychological Counseling,” and synonyms. We adapted the syntax for the specificities of each database (see Supplementary material 5 for all the information on the literature search). Next, we used the backward (the works cited in the selected articles) and forward search (the studies that cited the selected articles) to identify any further studies. Our systematic review considered the following inclusion criteria: (i) studies concerning the Italian university student population; (ii) studies carried out during the COVID-19 pandemic period; (iii) studies that have carried out/delivered an assessment and one or more psychological intervention(s). The exclusion criteria were set as follows: (i) Scientific publications such as editorials, letters, and case reports; (ii) studies that have used only qualitative assessment methods; (iii) studies that used only unvalidated psychological scales. We focused on university students because university education is a transition period characterized by critical challenges for students (e.g., career choice, emancipation, and financial self-sufficiency; Arnett et al., 2014; Amerio et al., 2022). Furthermore, the period of university study coincides with the years in which the risk of onset of psychiatric disorders reaches its peak (75% of all lifetime mental disorders have their onset prior to the age of 24; Kessler et al., 2005). Mental disorders during this period can be associated with adverse effects on the development of young people, including worsening academic performance and dropout from university (Scott et al., 2016; Bruffaerts et al., 2018). Furthermore, we focused on the Italian context as Italy was the first in Europe to face the consequences of the pandemic and implement rigorous measures to contain the spread of the virus. The measures taken to fight the virus in countries worldwide have been different and adapted to the specific national context, which has led to important differences in programs to contain the spread of the virus in different countries (Gavosto and Romano, 2021).

### 2.2 Study selection and data extraction

**Figure 1:**
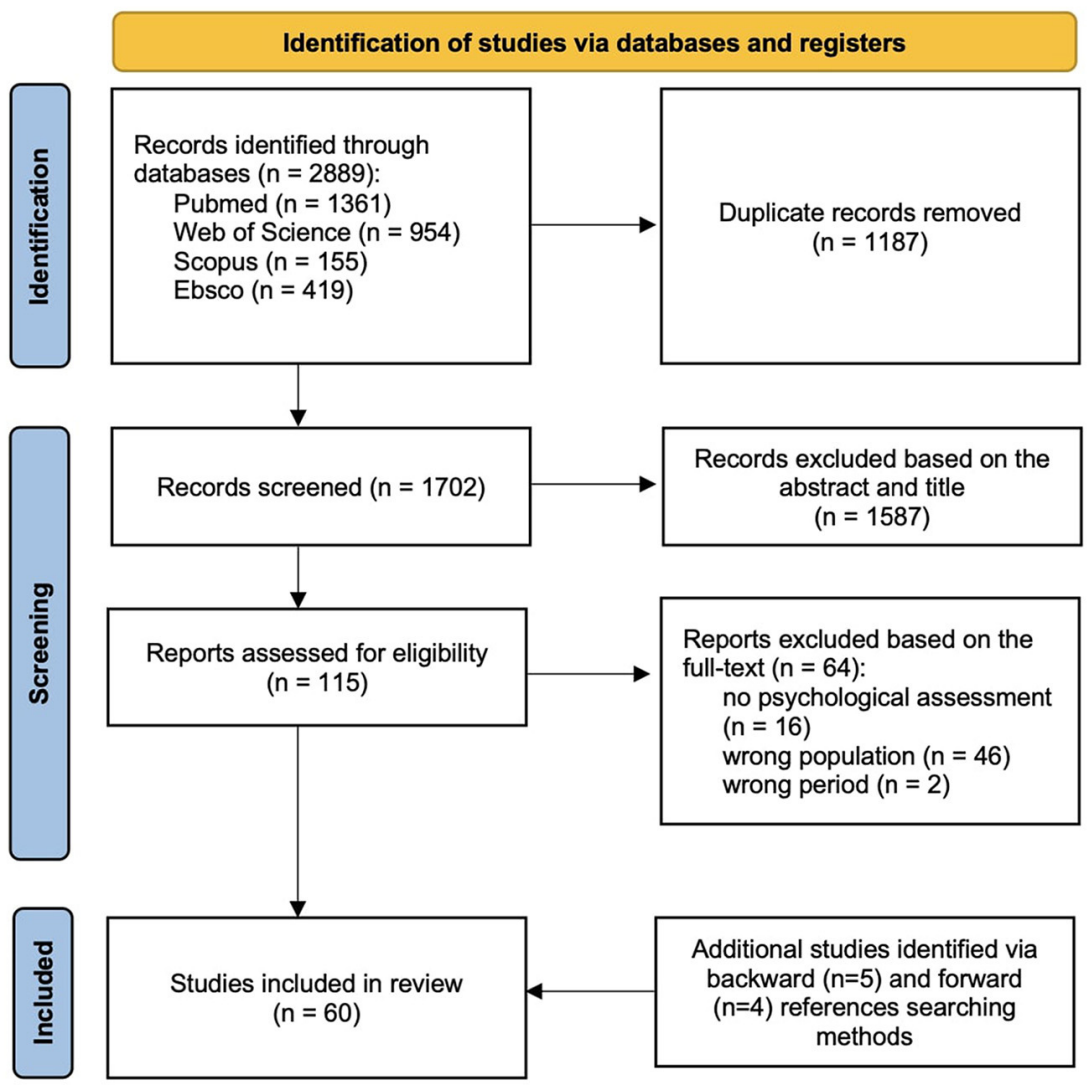
PRISMA flowchart of the literature search and screening process.

The screening process was performed using the digital tool “Rayyan” (Ouzzani et al., 2016). After removing duplicates, two authors performed the screening step independently (G.S., E.T.). This first step was based on the titles and the abstracts of each record yielded by the literature search. According to the eligibility criteria presented above, irrelevant studies were excluded. Then, after the first screening step, the same investigators read the full text of the remaining articles. For the letter, the same eligibility criteria were used for the inclusion/exclusion of the articles. During both phases of the study's selection, disagreements were resolved by discussion to find a consensus. Of the 1,702 studies initially retained, 60 publications met the eligibility criteria (Figure 1).

It should be noted that nine studies (Amatori et al., 2020; Biondi et al., 2021; Busetta et al., 2021; Di Consiglio et al., 2021; Cofini et al., 2022; Marzilli et al., 2022; Quintiliani et al., 2022; Alesi et al., 2023; Bassi et al., 2023) were added after backward and forward searching methods. For articles suitable for inclusion in this systematic review, the second and the third authors extracted data related to study characteristics (the number of participants, the types of assessment, the period of data collection, the instruments used for the assessment, the interventions, and the main findings reported). See Supplementary Table 1 (studies that investigated the psychological wellbeing of the general university student population) and Supplementary Table 2 (studies that investigated the psychological wellbeing of students attending university counseling services) for a description of the studies included in this systematic review. We decided to divide the studies into two sub-groups because students requiring psychological support have a higher psychological risk profile with more significant psychological distress than the general university student population (Riva Crugnola et al., 2021). In Supplementary Tables 1, 2, we have reported the period in which the assessment was carried out, dividing the phases of the pandemic into two macro-periods: (i) lockdown period and (ii) pandemic period. The period indicated as “lockdown” goes from the closure of all non-essential commercial activities and the imposition of home confinement on the population due to the spread of COVID-19 in Italy (March 2020) up to the first easing of restrictive measures (May 2020). This period was characterized by very restrictive measures for the population and the same for the entire Italian territory. The second period, indicated as the “pandemic,” includes the second and third phases of the pandemic in Italy following the first lockdown phase. This period was characterized by less restrictive home confinement measures and the gradual reopening of non-essential commercial activities. At this stage, the restrictions were often heterogeneous between the Italian regions due to their classification into three risk scenarios. Finally, in Supplementary Table 3, we reported additional information on studies included in the systematic review.

### 2.3 Risk-of-bias assessment

Our systematic review included several articles with different study designs. For this reason, we employed various tools to evaluate the quality of the evidence and the potential for bias. We applied the revised Cochrane Risk-of-Bias tool for randomized trials (RoB 2 – Higgins et al., 2016; Sterne et al., 2019). Furthermore, we used two different versions of the Newcastle-Ottawa Scale (NOS – Peterson et al., 2011) based on the study type: cohort studies (NOS – C) and cross-sectional studies (NOS – CS). Specifically, our systematic review included 3 randomized controlled trials (Cozzolino et al., 2021a; Celia et al., 2022a; Malighetti et al., 2023), 14 longitudinal cohort studies (Baiano et al., 2020, 2022; Bussone et al., 2020; Parola et al., 2020; Gabrielli et al., 2021; Meda et al., 2021; Pisano et al., 2021; Tinella et al., 2021; Celia et al., 2022b; Ierardi et al., 2022; Busetta et al., 2023; Cerutti et al., 2023; Conti et al., 2023; Renati et al., 2023), and 42 cross-sectional studies (Amatori et al., 2020; Cellini et al., 2020; Giusti et al., 2020, 2021; Savarese et al., 2020; Somma et al., 2020; Vitale et al., 2020; Abenavoli et al., 2021; Biondi et al., 2021; Burro et al., 2021; Busetta et al., 2021; Calandri et al., 2021; Commodari et al., 2021; De Pasquale et al., 2021a,b; Di Consiglio et al., 2021; Fornili et al., 2021; Generali et al., 2021; Marelli et al., 2021; Ranieri et al., 2021; Romeo et al., 2021; Villani et al., 2021; Viselli et al., 2021; Amerio et al., 2022; Bottaro and Faraci, 2022; Calati et al., 2022; Carletto et al., 2022; Carpi et al., 2022; Cofini et al., 2022; Comparcini et al., 2022; Concerto et al., 2022; Giangrasso et al., 2022; Guidotti et al., 2022; Loscalzo and Giannini, 2022; Manfredi, 2022; Marzilli et al., 2022; Quarta et al., 2022; Quintiliani et al., 2022; Zurlo et al., 2022a,b; Alesi et al., 2023; Bassi et al., 2023). The work conducted by Lo Moro et al. (2022) included two studies within the same article: a longitudinal cohort study and a cross-sectional one. Hence, we evaluated Lo Moro et al. (2022) twice, with the NOS – C and the NOS – CS, respectively. The evaluation of study quality was independently conducted by two authors (G.S., E.T.), who calculated percent agreement and Cohen's K (Cohen, 1960), obtaining a percentage of agreement P(a) = 97% and a Cohen's K of 0.84—an almost perfect agreement level. Any discrepancies or concerns were subsequently deliberated with the other authors. All studies obtained a satisfactory risk of bias: no concerns and high risk of bias were highlighted, and the studies were judged to be at low risk of bias. See Supplementary Tables 4, 5, and Supplementary Figure 4 for each study's risk of bias scores included in the systematic review.

## 3 Results

### 3.1 General results

The process through which the studies were included in the systematic review is described in the PRISMA flowchart (Figure 1). The initial search provided 2,889 studies potentially relevant to the present systematic review. Of these, 1,361 were identified from Pubmed, 954 from Web of Science, 155 from Scopus, and 419 from Ebscohost (APA PsycInfo). The duplicates were removed (*N* = 1,187), and 1,702 entries were screened. After screening the titles and abstracts, 1,587 articles were removed, leaving 115 articles to assess eligibility criteria. After reviewing the full texts, a further 64 were excluded. As shown in Figure 1, 64 articles were excluded for the following reasons: (i) no psychological assessments were performed (*n* = 16); (ii) no university students were assessed (*n* = 46); iii) the assessment was not carried out during the period of the COVID-19 pandemic (*n* = 2).

In addition to the 51 studies obtained through this selection process, we identified and included nine studies via backward and forward reference searching methods. This selection procedure supplied 60 articles suitable for inclusion in this systematic review. The data extracted from the 60 studies are summarized in Supplementary Table 1 (summary of psychological assessment findings in general university students' population) and Supplementary Table 2 (summary of psychological assessment/intervention findings in university students attending counseling services). Supplementary Table 1 contains forty-nine studies, while Supplementary Table 2 contains eleven. All studies delivered the assessment and interventions online during the lockdown and the subsequent pandemic period. Half of the forty-three studies that reported cross-sectional data investigated the psychological condition of students during the lockdown period, while the others delivered the assessment during the subsequent pandemic period. Five of these studies also collected the data before the lockdown period, while in four studies, participants were also asked to report their psychological state before the lockdown or pandemic period investigated. Six of the fifteen longitudinal cohort studies assessed the psychological wellbeing of Italian university students before and during the pandemic. Differently, four studies assessed it before and during the lockdown. The others (5 studies) delivered at least one psychological intervention during the pandemic.

Two randomized controlled trials provided at least one psychological intervention during the pandemic, while the other was during the lockdown period. 47.8% of the universities involved in the studies included in this review are from northern Italy (see Supplementary Table 3). Consistently, students from northern universities are the most represented (50.2%), followed by those studying in central Italy (34.71%), and finally, students from southern and island universities are the least numerous (15.04%). In almost all studies, the participants are mainly women. Of the six studies in which there are more male participants (Amatori et al., 2020; Baiano et al., 2020; Parola et al., 2020; Amerio et al., 2022; Concerto et al., 2022; Manfredi, 2022), only one (Concerto et al., 2022) have a percentage of women lower than 40%, while the remaining five have percentages of men only slightly higher than women. Most studies assess students from multiple departments. Instead, eight studies focus only on medical and nursing students (see Supplementary Table 3).

**Figure 2:**
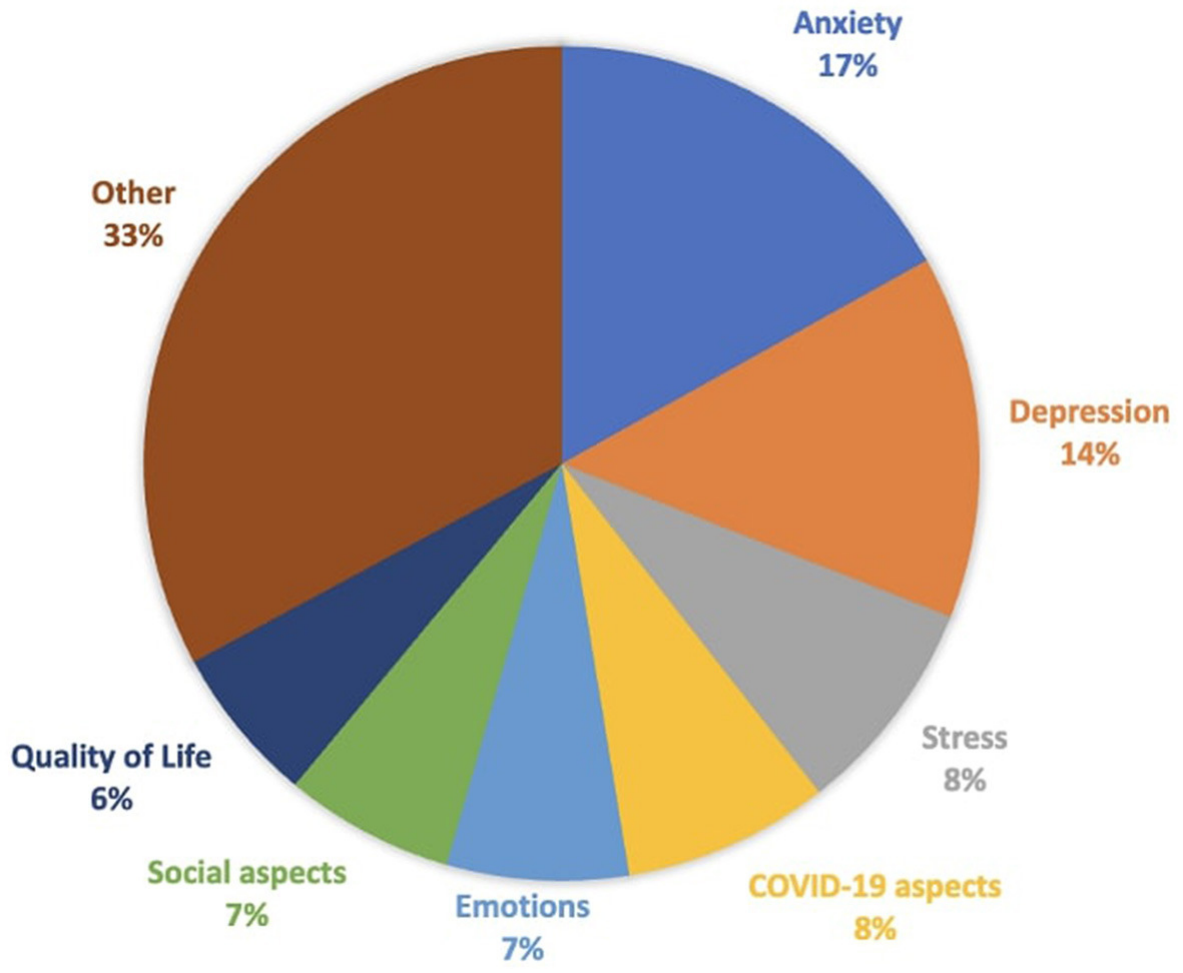
Percentage of the most psychological dimensions investigated in university students during the pandemic. The percentage is calculated using the total number of assessment tools in all studies.

**Figure 3:**
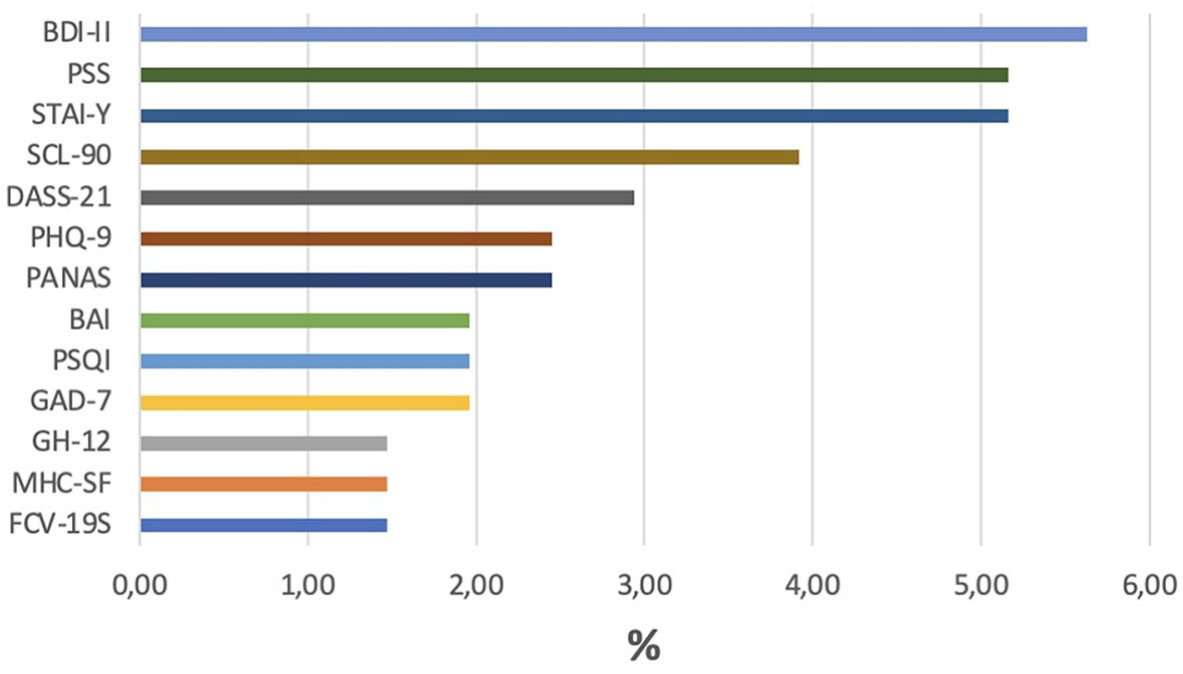
Percentage of assessment tools most used during the pandemic. The percentage is calculated using the total number of assessment tools in all studies.

As shown in Figure 2, the psychological dimensions most investigated by studies during the pandemic are anxiety (16.9%), depression (14%), stress (8%), aspects related to COVID-19 (e.g., fear and stress related to the pandemic), emotions (7%), social aspects (7%), and quality of life in general (6%). The “other” category contains 33% of all assessments considered in this review. This category includes studies that focused on psychological distress (5.16%), psychiatric symptoms other than depression and anxiety (e.g., symptoms of eating disorders, suicidal tendencies, smartphone, and internet addiction, 4.76%), and the quality of sleep (3.76%). Other psychological dimensions investigated, such as resilience, self-efficacy, self-esteem, metacognition, attitude to the future, coping, attachment, and personality traits, were explored by < 3% of the total assessment tools used in the studies. Finally, the most assessment instruments used are reported in Figure 3 (Beck Depression Inventory-II, BDI- II, 5.63%; State-Trait Anxiety Inventory, STAI-Y, 5.16%; Perceived Stress Scale, PSS, 5.16%; Symptom Check-List Item Revised, SCL-90-R, 3.92%; Depression, Anxiety and Stress Scale-21, DASS-21, 2.94%; Positive and Negative Affect Schedule, PANAS, 2.45%; Patient Health Questionnaire 9, PHQ-9, 2.45%; Beck Anxiety Inventory, BAI, 1.96%; Pittsburg Sleep Quality Index, PSQI, 1.96%; Seven-Item Generalized Anxiety Disorder, GAD-7; 1.96%; General Health Questionnaire, GH-12; 1.47%; Mental Health Continuum-Short Form, MHC-SF, 1.47%; Fear of COVID-19, FCV-19S, 1.47%).

### 3.2 Main findings in the wellbeing of the university student population

This literature review shows an adverse effect of the COVID-19 pandemic on the psychological wellbeing of Italian university students (i.e., Bussone et al., 2020; Commodari et al., 2021; Pisano et al., 2021; Viselli et al., 2021; Alesi et al., 2023). Studies mainly report increases in levels of depression (i.e., Conti et al., 2023), anxiety (i.e., Abenavoli et al., 2021), and stress (i.e., Lo Moro et al., 2022), both during the period of more significant lockdown restrictions and in the subsequent phase of the pandemic. Some longitudinal studies report an additive increase in the adverse effects on psychological wellbeing as the weeks pass during lockdown (Giusti et al., 2020; Parola et al., 2020; Zurlo et al., 2022a). However, during the subsequent pandemic phase, some psychological dimensions (i.e., depression) may improve compared to the lockdown's previous and more restrictive phase (Di Consiglio et al., 2021; Meda et al., 2021). Another result that emerges is that the students who have suffered most from the measures imposed by the pandemic are women students (Vitale et al., 2020; Busetta et al., 2021, 2023; Romeo et al., 2021; Villani et al., 2021; Viselli et al., 2021; Amerio et al., 2022; Carletto et al., 2022), and younger students (Fornili et al., 2021; Ranieri et al., 2021; Concerto et al., 2022). Although no studies included in this review directly compared students who sought university counseling services with Italian university students in general (but see Riva Crugnola et al., 2021), some studies (Baiano et al., 2020; Cellini et al., 2020; Busetta et al., 2021) reported a greater negative effect of the COVID-19 pandemic on fragile students (i.e., students with high levels of depression and anxiety). Finally, Ierardi et al. (2022) reported that students who sought college counseling services before the pandemic had similar levels of psychological distress as those who did so during the pandemic.

It has been suggested that medical students are more likely than students of other faculties to experience higher levels of anxiety regarding COVID-19 (Busetta et al., 2023). However, not all data confirm this view (e.g., Manfredi, 2022; Bassi et al., 2023). These inconsistent results could depend on the fact that the studies that include healthcare students are heterogeneous by year of course and type of specialization taken into consideration. University students have reported significant difficulties related to distance learning during the pandemic, such as reduced concentration ability and problems completing the thesis and internship activities (Generali et al., 2021; Giusti et al., 2021). More severe psychological symptomatology was positively correlated with worse academic performance (Amerio et al., 2022). The inability to attend university, distance from colleagues, and career uncertainty were associated with increased anxiety (Villani et al., 2021; Comparcini et al., 2022; Busetta et al., 2023). Furthermore, Calandri et al. (2021) reported that the worsening of learning abilities was related to depressive symptoms through the mediating effect of academic self-efficacy (Calandri et al., 2021). Fear of COVID-19 and worry about contracting the virus appear to be important factors in determining higher levels of anxiety and negative changes in mood and eating behavior, both during the lockdown and during the pandemic (De Pasquale et al., 2021a,b; Generali et al., 2021). Lastly, internalizing personality traits and immature defense mechanisms were both risk factors for depression, anxiety, and stress symptoms during the lockdown (Biondi et al., 2021).

On the contrary, a healthy lifestyle (i.e. doing physical activity, healthy nutrition) (Amatori et al., 2020; Fornili et al., 2021; Villani et al., 2021), a healthy relational environment (family, peer, and university) (Calandri et al., 2021; Carletto et al., 2022), satisfaction with the quality of e-learning (Giusti et al., 2021; Cofini et al., 2022), and the possibility of benefiting from external spaces and contact with nature (Busetta et al., 2021; Quarta et al., 2022), are all factors reported as positive for mental health both during the lockdown and during the pandemic. Adaptive coping strategies were protective factors for managing COVID-19 stressors (Zurlo et al., 2022b), and high levels of resilience predicted lower levels of psychological distress after a year of the pandemic (Renati et al., 2023). Furthermore, the study by Giangrasso et al. (2022) reported that high levels of mattering and self-esteem are crucial for adequate life satisfaction during the lockdown.

Almost all studies conducted on students who requested psychological counseling from university services have focused on the effectiveness of online psychological intervention (see Supplementary Table 2). These works show that five sessions of online psychological interventions (Tinella et al., 2021) with psychodynamic (Ierardi et al., 2022; Cerutti et al., 2023) and strategic (Celia et al., 2022b) approaches, and the Brain Wave Modulation Technique (BWM -T) (Cozzolino et al., 2021b; Celia et al., 2022a), are effective in reducing perceived stress, negative affects, depressive and anxious symptoms, and in improving different aspects of students' psychological and emotional wellbeing (i.e., self-efficacy, emotional regulation, positive affect) both during the lockdown and the pandemic. Furthermore, Gabrielli et al. (2021) and Malighetti et al. (2023) report decreases in levels of anxiety and perceived stress using a healthy coping chatbot (“Atena,” 4 weeks of use) and improvements in psychological wellbeing through self-help virtual reality interventions (six sessions for 3 weeks), respectively. However, Ierardi et al. (2022) reported that the face-to-face intervention delivered before the pandemic was more effective than the online intervention delivered during the pandemic. While the online intervention improved levels of depression, anxiety, and obsessive-compulsive symptoms, differently from face-to-face intervention, it was not effective in improving levels of life satisfaction (Ierardi et al., 2022). Although none of these studies directly investigated the effects of university psychological counseling on academic performance, worse psychological symptomatology has been reported to be a strong predictor of poor academic performance (Giusti et al., 2021). Finally, it is important to note that some university psychological counseling services have integrated specific interventions to improve academic performance (e.g., workshops on study methodologies in small groups of students in Savarese et al., 2020).

## 4 Discussion

A total of 60 articles were included in the present systematic review, sourced from various academic databases and through backward and forward reference searching methods. The review encompasses a comprehensive set of studies, including both cross-sectional and longitudinal designs, as well as randomized controlled trials. This diversity in study designs enriches the understanding of the psychological dynamics among university students during the pandemic.

The studies included in the review primarily focused on assessing the psychological wellbeing of university students during the COVID-19 pandemic, with a notable emphasis on conducting assessments and interventions online during lockdown periods. The temporal distribution of studies provides insight into the evolving nature of the pandemic's impact on students' mental health: while some studies assessed psychological conditions during the lockdown period, others examined the subsequent pandemic period, offering a nuanced understanding of students' experiences over time.

The geographical distribution of universities included in the studies highlights a concentration of research in northern Italy. This regional focus may reflect varying degrees of COVID-19 impact across different regions and underscores the need for comprehensive, region-specific interventions and support projects. This finding also calls for the urgent need to better investigate the mental health of students in southern Italy, also considering the economic and structural gaps between northern and southern Italy and the impact these can have on psychological health and adult identity development (Piumatti et al., 2016).

The results also identified specific demographic patterns. First, the predominance of female participants across most studies raises important considerations regarding gender-specific mental health needs and disparities in help-seeking behaviors. In line with this predominance and with the general international literature (Almeida et al., 2020; Qiu et al., 2020), studies showed that women and younger students appeared to be disproportionately affected by the pandemic measures, experiencing higher levels of psychological distress compared to their male and older counterparts. These results can be considered in line with the significant worsening in quality of life reported in female university students during the pandemic (Sulla et al., 2023). Furthermore, young students may have suffered more from the negative consequences of the pandemic due to less adaptive strategies than adults, which would consequently make them more at risk of developing psychological distress during a stressful event (Schilling and Diehl, 2015; but see also Koch and Park, 2022). This disparity highlights the need to pay particular attention to the most vulnerable categories (Saifee et al., 2021) and the need for targeted interventions tailored to the specific needs and vulnerabilities of these groups (Gatto et al., 2022; Koch and Park, 2022). In addition, studies also showed that medical students, in particular, may experience heightened levels of anxiety related to COVID-19. Although results are not consistent across studies, this result appears in line with the international literature (Mittal et al., 2021). Overall, the results suggested that understanding the unique stressors faced by vulnerable student populations is essential for developing targeted interventions and support services.

The reviewed studies used diverse array of assessment instruments, underscoring the complexity of measuring, and understanding psychological wellbeing during times of crisis but also showing a lack of homogeneity in methodology. Overall, the most investigated psychological dimensions during the pandemic included anxiety, depression, stress, COVID-19-related fears and stress, emotions, social aspects, and general quality of life. These findings widely underscored the multifaceted nature of the pandemic's impact on students' mental health and highlight the need for tailored interventions also addressing diverse psychological domains. Specifically, in line with the international literature (Alkhamees et al., 2020; Cao et al., 2020; Savage et al., 2020; Wang et al., 2020; Wathelet et al., 2020; Chen and Lucock, 2022; Faisal et al., 2022), the studies highlighted a consistent increase in levels of depression, anxiety, and stress among university students during both the lockdown period and the subsequent phases of the pandemic. Some longitudinal studies indicate an exacerbation of adverse effects on psychological wellbeing as the duration of lockdown measures extended, emphasizing the cumulative stress experienced by students during prolonged periods of restriction. Several protective factors, including healthy lifestyle practices, social support, and adaptive coping strategies, emerged as mitigating factors against pandemic-induced psychological distress. From our point of view, the knowledge of risk and protective variables for mental health, even though referred to the COVID-19 period, might be useful in defining the areas on which the university counseling interventions can focus and in informing resilience-building interventions.

Regarding the studies that investigated the effectiveness of online psychological interventions, psychodynamic approaches as well as strategic interventions demonstrated effectiveness in reducing perceived stress, depressive and anxious symptoms, and improving emotional wellbeing among university students. This result is overall in line with the international literature (Son et al., 2020). However, the review also acknowledged variations in the effectiveness of online interventions compared to face-to-face modalities, suggesting nuanced approaches to intervention delivery.

While the systematic review provides valuable insights into the psychological impact of the COVID-19 pandemic on Italian university students, several limitations should be considered when interpreting the findings. The selection criteria employed in this review might lead to selection bias because studies failing to meet particular eligibility standards were excluded, thereby constraining the applicability of the results and disregarding significant subtleties in the psychological experiences of university students amid the pandemic. The studies included in the review exhibit heterogeneity in terms of study design, participant demographics, assessment tools, and intervention modalities. This diversity makes it challenging to synthesize findings and draw definitive conclusions across studies. Many of the included studies rely on self-report measures to assess psychological wellbeing, which may be susceptible to response bias and inaccuracies in reporting. Additionally, the lack of standardized assessment protocols across studies may compromise the validity and reliability of the findings. The review predominantly captures short-term effects of the pandemic on students' mental health, primarily during the lockdown and immediate post-lockdown periods. Long-term effects and trajectories of psychological wellbeing beyond the initial phases of the pandemic may not be adequately addressed. Moreover, it is important to underline that university counseling services constitute devices for the wellbeing and psychological health promotion, while these do not constitute health agencies and, therefore, do not have either the structure or the functions to actively combat serious situations. In this sense, it should be also clarified that universities do not always have standard and generalizable protocols on the management of serious cases that might help to create a more replicable and generalizable approaches within the different university settings. While the review highlights the effectiveness of online psychological interventions, the quality and rigor of the included intervention studies vary. Methodological limitations, such as small sample sizes, lack of control groups, and short follow-up periods, may limit the robustness of the evidence supporting intervention effectiveness. Addressing these limitations requires future research endeavors to adopt more rigorous methodologies, including larger sample sizes, longitudinal designs, and standardized assessment protocols. Efforts to explore eventual long-term psychological consequences of the pandemic and identify factors contributing to resilience and wellbeing among university students are warranted. Additionally, the results obtained from the present review are only partially generalizable since, despite the similar emergency characteristics experienced by different countries during COVID-19 pandemic, each country varied greatly both in terms of spread of the virus as well as of measures adopted to limit the infection. These variations have inevitably produced different conditions and measures also within the university settings, therefore, this aspect calls for caution in the interpretation of results.

## 5 Conclusion

The broad spectrum of research designs reviewed significantly enriched our comprehension of the psychological dynamics experienced by university students during the COVID-19 pandemic. The temporal distribution of studies offered insights into the evolving nature of the pandemic's impact on students' mental health, spanning from lockdown periods to subsequent phases. Geographically, a concentration of research in northern Italy highlighted the need for region-specific interventions and underscored the urgency to investigate the mental health of students in southern Italy, considering economic and structural disparities. Demographically, female, and younger students exhibited higher levels of psychological distress, emphasizing the importance of tailored interventions for vulnerable groups. Additionally, medical students showed heightened anxiety related to COVID-19, necessitating targeted support. The diverse array of assessment instruments used in the reviewed studies highlighted the complexity of measuring psychological wellbeing during crises, yet also revealed a lack of methodological homogeneity. Overall, studies indicated elevated levels of depression, anxiety, and stress among university students during lockdowns and subsequent pandemic phases, with longitudinal studies highlighting the cumulative stress over prolonged periods of restriction. Protective factors such as healthy lifestyle practices and social support emerged as crucial mitigating factors against pandemic-induced distress, informing resilience-building interventions within university settings. These findings broadly underscore the importance of ongoing monitoring and support for students' mental health in higher education context even beyond the challenging times. In terms of intervention, online psychological interventions, including psychodynamic and strategic approaches, demonstrated effectiveness in reducing stress and improving emotional wellbeing among students, suggesting the need to even more implement and potentiate these within university settings. Despite their effectiveness, we cannot prove specifically the extent of their impact, the duration, and the differences there may be between one type of intervention rather than another. Therefore, there emerges a need to strengthen and improve the quality standards of such interventions and their evaluation through rigorous experimental or quasi-experimental research design, such as process and outcome studies, in order to show the true extent of their function and effects. Furthermore, variations in effectiveness compared to face-to-face modalities also suggest the need for nuanced intervention delivery methods. In conclusion, the findings underscore the importance of prioritizing mental health support services within university settings and the critical need for comprehensive evidence-based mental health interventions, region-specific support systems tailored to the unique needs and challenges faced by university students. Providing accessible and effective mental health resources, including online interventions and counseling services, is crucial for mitigating the adverse effects of the pandemic on students' psychological wellbeing also in the long-term period. We argue that these issues become even more urgent considering the Global Goals of the 2030 Agenda for Sustainable Development that, within the goals number 3 and 4, stresses the need to promote the mental health and wellbeing of general population, especially youths, as well as to provide quality, equitable, and inclusive education for all students. In line with this, our findings call for the need to increase healthcare financing in university settings to guarantee and defend the student's health and wellbeing also promoting lifelong learning opportunities for all.

## Data availability statement

The original contributions presented in the study are included in the article/Supplementary material, further inquiries can be directed to the corresponding author.

## Author contributions

GC: Conceptualization, Data curation, Formal analysis, Investigation, Methodology, Project administration, Supervision, Writing – original draft, Writing – review & editing. GS: Conceptualization, Data curation, Formal analysis, Investigation, Methodology, Project administration, Writing – original draft, Writing – review & editing. ET: Data curation, Formal analysis, Investigation, Methodology, Writing – original draft, Writing – review & editing. FT: Methodology, Writing – original draft, Writing – review & editing, Investigation. MC: Funding acquisition, Project administration, Supervision, Writing – original draft, Writing – review & editing.
