# Supplementary material for: Psychological wellbeing of Italian students and clinical assessment tools at university counseling services during the COVID-19 pandemic: a systematic review
Source: Front Psychol. 2024 Aug 13;15:1388419. doi: 10.3389/fpsyg.2024.1388419 (PMC11347296; doi:10.3389/fpsyg.2024.1388419)
Supplement: Supplementary file 1 [file Table_1.DOCX]

Supplementary Material

**Psychological well-being of Italian university students and clinical assessment tools at university counseling services: A Systematic Review**

Supplementary Material 1 – Table 1 – Summary of psychological assessment findings in general university students’ population **…page 2**

Supplementary Material 2 – Table 2 – Summary of psychological assessment/intervention findings in university students attending counseling services**…page 12**

Supplementary Material 3 – Table 3 – Additional information of studies included in the systematic review **…page 15**

Supplementary Material 4 – Table 4 and 5, and Figure 4 – Risk of bias in each study **…page 21**

Supplementary Material 5 – Literature search and results **…page 25**

**Supplementary Material 1 – Table 1 – Summary of psychological assessment findings in general university students’ population**

Table 1. Summary of psychological assessment findings in general university students’ population.

| **Authors (year)** | **Participants (N)** | **Type of Assessment** | **Instruments** | **Main findings** |
| --- | --- | --- | --- | --- |
| Abenavoli et al. (2021) | (354) | Web Assessment (referring to before and during the lockdown) | Short Anxiety Inventory (SHAI) | Anxiety levels increased by 31.26% during the lockdown |
| Alesi et al. (2023) | (1028) | Web Assessment (during the pandemic) | Personality Inventory (PI) State-Trait Anxiety Inventory (STAI-Y) General Self-Efficacy Scale Academic Motivation Scale (AMS-C 28) Fear of COVID-19 (FCV-19S) | Students suffered mainly from anxiety (46.1%), mood alteration (43.5%), and insomnia (35%) as a negative consequence of the COVID-19 pandemic. Moreover, results showed two clusters of students having a Maladaptive Academic Profile (44.4% - higher levels of neuroticism, higher dispositional and situational anxiety, and fear of COVID-19, and lower self-efficacy and academic motivation; more physical or mental health problems and help requests) or an Adaptive Academic Profile (55.6%) |
| Amatori et al. (2020) | (176) | Web Assessment (during the lockdown) | Positive and Negative Affect Schedule (PANAS) Patient Health Questionnaire 9 (PHQ-9) 12-Item Short Form Health Survey (SF-12) | 13% of males and 19% of females reported moderate to severe symptoms of depression. Depression and quality of life were directly and inversely associated with cereals, legumes, and low-fat meat intake. Exercise led to healthier nutritional choices |
| Amerio et al. (2022) | (8177) | Web Assessment (during the lockdown) | Nine-item Patient Health Questionnaire (PHQ-9)  Seven-item Generalized Anxiety Disorder (GAD-7)  Seven-item Insomnia Severity Index (ISI)  Barratt Impulsiveness Scale-11 (BIS-11) | 12.8% of participants reported depressive symptoms, 25.6% anxiety, 8.7% insomnia, and 10.6% impulsive tracts. Higher proportions of symptoms among females than males |
| Baiano et al. (2020) | (25) | In-person Assessment before (T0) the lockdown and Web Assessment at the end (T1) of the lockdown | Penn State Worry Questionnaire (PSWQ)  Anxiety Sensitivity Index-3 (ASI-3)  Mindful Attention Awareness Scale (MAAS) | High worry was reported by 60% of responders at T0 and 68% at T1. At T1, trait mindfulness was inversely related to worry and fear of mental health. Hight worriers participants showed at T1 a significant increase in anxiety sensitivity and fear of mental health compared to low worriers participants |
| Baiano et al. (2022) | (69) | In-person Assessment before (T0) the lockdown and Web Assessment after a year (T1) of the pandemic | Interpersonal Reactivity Index (IRI)  Empathy Quotient (EQ)  Reading the Mind in the Eyes Test  Faux Pas Test | Higher scores at T1 than T0 in IRI Perspective Taking, IRI Fantasy, and Empathic Concern. Higher scores in empathy quotient (EQ) and emotional reactivity and lower scores in social skills at T1 compared to T0 |
| Bassi et al. (2023) | (1799) | Web Assessment (during the lockdown) | Mental Health Continuum Short-Form (MHC-SF) Need for Closure Scale (NFC) | Healthcare students reported higher mental health than students attending social sciences and humanities courses. Moreover, results showed that the NFC dimensions of the need for order and decisiveness were positively associated with all mental health components; in contrast, the need for predictability, intolerance for ambiguity, and close-mindedness were negatively associated with all mental health components |
| Biondi et al. (2021) | (1047) | Web Assessment (during the lockdown) | Personality Inventory for the DSM-5 – Brief Form (PID-5-BF) Defense Style Questionnaire-40 (DSQ-40) Depression, Anxiety and Stress Scale-21 (DASS-21) | Results showed that immature defense mechanisms and internalizing personality traits (i.e., detachment, negative affect, psychoticism) were risk factors for depression, anxiety, and stress symptoms |
| Bottaro & Franci (2022) | (353) | Web Assessment (during the pandemic) | Multidimensional Assessment of COVID-19-Related Fears (MAC-RF)  Intolerance of Uncertainty Scale-Short Version (IUS12) Life Orientation Test-Revised (LOT-R) AMOS Test (‘QC’ & ‘QAR’) | Positive correlations between fear of COVID-19 and intolerance of uncertainty and between intolerance of uncertainty and optimism. Negative correlation between fear of COVID-19 and optimism. Older age was linked to higher levels of optimism |
| Burro et al.  (2021) | (2987) | Web Assessment (during the lockdown) | Robust-Pandemic Coping Scale (R-PCS) | Students had higher scores in Adjustment and Proactivity compared to Despair and Aversion. Males scored lower than females for Despair, Adjustment, and Proactivity and higher scores for Aversion. Proactivity scores were lower for younger students than for older students |
| Busetta et al. (2021) | (4379) | Web Assessment (during the lockdown) | State-Trait Anxiety Inventory (STAI-Y) | Women, compared to men, are almost 20% more likely to experience a high level of state anxiety, which decreases to 15% for fragile students (high level of state anxiety with state score higher than trait score). Students who benefit from outdoor spaces (such as a garden or a terrace) are less likely to show high anxiety levels |
| Busetta et al. (2022) | (317) | Web Assessment at the beginning (T0) and after a year (T1) of the pandemic | State-Trait Anxiety Inventory (STAI-Y) | Higher anxiety levels at the beginning of the pandemic predicted a higher increase in anxiety after one year of COVID-19-related restrictions. Compared to men, women were more likely to experience higher anxiety levels after one year of the pandemic. University students from medical areas were more likely to experience a higher level of anxiety. Sleeping either less or more than habitual levels positively correlated with anxiety. The students’ frequency of using social networks was positively correlated with anxiety levels |
| Bussone et al. (2020) | (68) | Web Assessment before (T0) and during (T1) the lockdown | Symptom Check-List Item Revised (SCL-90-R)  Perceived Stress Scale (PSS)  State-Trait Anxiety Inventory (STAI-Y)  Relationship Questionnaire (RQ)  Parental Bonding Instrument (PBI) | Students showed higher depression, phobic anxiety, perceived stress, and psychological distress scores at T1 compared to T0. Students with secure attachment and high levels of parental care showed increased levels of state anxiety and perceived stress at T1 compared to T0 |
| Calandri et al.  (2021) | (296) | Web Assessment (during the lockdown) | Center for Epidemiologic Studies Depression Scale (CESD-10) Multidimensional Negative Regulatory Emotional Self-Efficacy Scale  Multidimensional Scales of Perceived Self-Efficacy (MSPSE) | 67 % of participants reported significant depressive symptoms. Depressive symptoms were directly related to the worsening of family relationships. Contagion concerns and worsening of learning skills were related to depressive symptoms through the mediating effect of emotional self-efficacy and academic self-efficacy, respectively |
| Calati et al. (2022) | (1586) | Web Assessment (during the pandemic) | Interpersonal Needs Questionnaire (INQ) Acquired Capability of Suicide Scale-Fearlessness About Death (ACSS-FAD); Psychache Scale  State-Trait Anxiety Inventory (STAI-Y)  Beck Depression Inventory-II (BDI-II) Rosenberg Self-Esteem Scale (RSES) Visual Analogue Scale (VAS)  Pain Vigilance and Awareness Questionnaire (PVAQ); Self-Awareness Questionnaire (SAQ) UCLA Loneliness Scale; Multidimensional Scale of Perceived Social Support (MSPSS) | History of suicidal planning and suicide attempts in participants were strongly associated with higher fearlessness about death and higher psychological pain |
| Carletto et al. (2022) | (1329) | Web Assessment (during the pandemic) | Generalized Anxiety Disorder Scale (GAD-7) Beck Depression Inventory-II (BDI-II) Perceived Stress Scale (PSS) | 47.8% of participants reported anxiety symptoms, 52.1% depression, and 28.4% severe perceived stress. The factors associated with mental health symptoms were being a woman, a family history of psychiatric disorders, living off-site, competitive/hostile climates and unsatisfying friendships among classmates, poor relationships with cohabitants, negative judgment of medical school choice, fear of COVID-19 infection, feelings of loneliness, distressing existential reflections, and a worsening psychological condition related to the pandemic |
| Carpi et al. (2022) | (1279) | Web Assessment (during the pandemic) | Pittsburgh Sleep Quality Index (PSQI)  10-item Perceived Stress Scale (PSS-10)  Short Form-12 questionnaire (SF-12 - HRQoL) | 65% of participants reported poor sleep quality, and 55% reported insomnia symptoms. Poor sleep quality and relevant insomnia symptoms were associated with higher distress and a worse physical and mental health-related quality of life |
| Cellini et al. (2020) | (809) | Web Assessment (referring to before and during the lockdown) | Pittsburg Sleep Quality Index (PSQI) Depression Anxiety Stress Scales (DASS-21) | Quality of sleep decreased during the lockdown, especially in participants with higher DASS-21 score |
| Cofini et al. (2022) | (471) | Web Assessment (during the pandemic) | E-Learning Satisfaction Scale Health-Related Quality of Life (HRQoL) General Health Questionnaire (GH-12) Brief Cope Scale (BC) | Results showed that the e-learning satisfaction score was inversely correlated with stress. Students with a high level of social presence had a high level of satisfaction with e-learning |
| Commodari et al.  (2021) | (655) | Web Assessment (during the lockdown) | Risk Perception of Infectious Disease Questionnaire;  Interpersonal Adaptation Questionnaire (QAI) Mesure du Stress Psychologique (MSP) Metacognitive Skills Scale (MSS) | 40% of students reported greater feelings of sadness (51.3%), nervousness (64.6%), and irritability (57%) than usual, with increased ruminations (70.9%). Furthermore, difficulty concentrating (55.9%) and sleeping (54.5%) were also reported. |
| Comparcini et al.  (2022) | (842) | Web Assessment (during the pandemic) | Self-Rating Anxiety Scale (SAS)  11-item Altered Student Study Environment Tool (ASSET) | 88.5% of students reported an Anxiety Level equal to or over the threshold. 19 % of the variance in anxiety was explained by students’ academic concerns relating to the altered study environments. The factor “grade attainment” was a positive and highly significant predictor of students’ anxiety in the total sample |
| Concerto et al.  (2022) | (399) | Web Assessment (during the pandemic) | Depression Anxiety Stress Scale short version (DASS-21)  Temperament Evaluation of Memphis, Pisa and San Diego Autoquestionnaire (TEMPS-A) Mental Health Continuum Short Form (MHC-SF) | DASS-21 scores showed a prevalence of severe and extremely severe levels of stress (40.6%), anxiety (87.5%), and depression (66.9%). Increasing age was associated with an increased likelihood of exhibiting a positive mental state |
| Conti et al.  (2023) | (177) | Web Assessment before (T0) and during (T1) the pandemic | 20-item UCLA Loneliness Scale – version 3 9-item Patient Health Questionnaire (PHQ-9)  7-item Generalized Anxiety Disorder Scale (GAD-7)  15-item Patient Health Questionnaire (PHQ-15) 20-item Toronto Alexithymia Scale (TAS-20) | Students reported worsening depression, anxiety, and alexithymia over time. In students with high loneliness, these effects were more prominent |
| De Pasquale et al. (2021) (a) | (194) | Web Assessment (during the pandemic) | Fear of COVID-19 Scale (FCV-19S)  Perceived Vulnerability to Disease (PVD)  State-Trait Anxiety Inventory (STAI)  Smartphone Addiction Scale - Short Version for Adolescents and Young Adults (SAS-SV) | Students showed a high risk of smartphone addiction, a moderate trait and state anxiety, and a moderate perceived vulnerability to disease. Fear of COVID-19 and trait anxiety appear to be the predictors of state anxiety and perceived vulnerability to disease |
| De Pasquale et al.  (2021) (b) | (469) | Web Assessment (during the pandemic) | Fear of COVID-19 Scale (FCV-19S)  Profile of Mood States (POMS)  Eating Disorder Inventory-2 (EDI-2)  Binge Eating Scale (BES) | Women were more exposed to fear of COVID-19, showing significantly higher levels of tension, fatigue, depression, and confusion and a higher total mood disturbance score than males. Fear of COVID-19 appears to be a predictor of mood changes and disordered eating behavior |
| Fornili et al.  (2021) | (14028) | Web Assessment (during the lockdown) | Hospital Anxiety and Depression Scale (HADS) | 20% of participants reported severe levels of anxiety and depression. Younger individuals and females had higher levels of psychological distress. Having a low income or low level of education in the family was related to higher levels of psychological distress; similar results were observed for sharing the house with old or disabled people. Performing physical activity during lockdown was associated with a lower risk of being severely anxious or severely depressed |
| Generali et al.  (2020) | (399) | Web Assessment (during the lockdown) | 7-item Generalized Anxiety Disorder (GAD-7) | 6.5% of the students showed severe anxiety levels. The GAD-7 score was positively correlated with concerns about contracting COVID-19 during daily and college activities |
| Giangrasso et al.  (2022) | (350) | Web Assessment (during the lockdown) | General Mattering Scale (GMS)  Anti-Mattering Scale (AMS)  Rosenberg Self-Esteem Scale (RSES)  Difficulties in Emotion Regulation Scale (DERS)  Hospital Anxiety and Depression Scale (HADS) Perceived Stress Scale (PSS)  Satisfaction with Life Scale (SWLS) | Feelings of not mattering were associated with lower life satisfaction and more significant reported difficulties in emotion regulation, stress, and distress. Mattering and self-esteem were unique predictors of life satisfaction levels during the lockdown |
| Giusti et al.  (2021) | (203) | Web Assessment (during the pandemic) | Beck Depression Inventory-II (BDI-II; 10 item)  Eyes Task (Reading the Mind in the Eyes Test-Revised) | 23.6% of students reported severe depressive symptoms. Negative correlation between depression and the overall subjective evaluation of distance learning |
| Guidotti et al. (2022) | Pre-pandemic group (78) Pandemic group (120) | In-person assessment before the pandemic (pre-pandemic group) and at the end of the pandemic (pandemic group) | Symptom Questionnaire (SQ)  P Stress Questionnaire (PSQ)  16 Personality Factor Questionnaire (16PF) | Higher levels in the global clinical scales of Anxiety, Depression, and Somatization in the pandemic group compared to the pre-pandemic group. High levels of somatic tension and emotional instability were predictive of levels of anxiety. High tension, low social boldness, and somatic complaints were predictive of depression levels |
| Lo Moro et al. (2022) | Study 1: longitudinal sample (121)    Study 2:  pre-pandemic group (283) pandemic group (422) | Web Assessment before (T0) and during (T1) the pandemic  Web Assessment before and during the pandemic | Beck Depression Inventory-II (BDI-II)  Perceived Stress Scale-10 (PSS-10)  Beck Depression Inventory-II (BDI-II)  Perceived Stress Scale-10 (PSS-10) | Higher levels of depressive symptoms and stress during the pandemic compared to before  Higher levels of depressive symptoms and stress in the pandemic group respect to pre-pandemic group |
| Loscalzo & Giannini  (2021) | (6075) | Web Assessment (during the lockdown) | Positive and Negative Affect Schedule (PANAS)  Depression Anxiety Stress Scales-21 (DASS-21)  Health Anxiety Questionnaire (HAQ) | Women, compared to men, have a higher level of agreement with both social isolation and quarantine measures. Value of health anxiety predicted higher levels of worries about having contracted the virus |
| Manfredi et al.  (2021) | (90) | Web Assessment (during the pandemic) | General Health Questionnaire (GHQ 12)  Maslach Burnout Inventory (MBI)  Subjective Happiness Scale (SHS)  Satisfaction with Life Scale (SWLS)  Coping Inventory for Stressful Situations (CISS) Brief Resilience Scale  State-Trait Anxiety Inventory (STAI) | Results showed high values of resilience and job satisfaction, a positive assessment of the support received from the work team, an articulate use of coping strategies, and good levels of happiness and satisfaction with life. However, widespread anxiety also emerged, which appears to be more attributable to concerns about professional evaluation (trainees in psychiatry and anesthesia–-resuscitation) rather than the pandemic itself |
| Marelli et al. (2021) | (307) | Web Assessment (referring to before and during the lockdown) | Pittsburgh Sleep Quality Index (PSQI)  Insomnia Severity Index (ISI)  Morningness-Eveningness Questionnaire (MEQ) Beck Anxiety Inventory (BAI)  Beck Depression Inventory-II (BDI-II) | Students reported worsening sleep quality and insomnia symptoms during the lockdown. A clinically significant somatic-affective and cognitive BDI score was found in 25.9% and 43.2% of students, respectively. A clinically significant BAI total score was found in 38.2% of students |
| Marzilli et al. (2022) | (410) | Web Assessment during the pandemic | Inventory of Parent and Peer Attachment (IPPA) COVID-19 Peritraumatic Distress Index (CPDI) Toronto Alexithymia Scale (TAS-20) Internet Addiction Test (IAT) | Female university students showed significantly higher peritraumatic distress symptoms due to COVID-19 than males. The results showed that the IAT scores were significantly and positively associated with the CPDI and TAS scores and negatively related to the scores of the IPPA |
| Meda et al.  (2021) | (161)  (197) | Web Assessment before (T0) and during the lockdown (T1)  Web Assessment before(T0) and after the lockdown (T1) | Beck Depression Inventory-II (BDI-II)  Beck Anxiety Inventory (BAI)  Obsessive-Compulsive Inventory-Revised (OCI-R) Eating Habits Questionnaire (EHQ)  Eating Disorder Inventory-3 (EDI-3) | BDI-2 median score was significantly higher in students during the lockdown than before the imposition of the lockdown  BDI-2 scores after the lockdown were similar to those observed before. BAI scores decreased after the lifting of the lockdown. Similarly, also the OCI-R scores were reduced after the lockdown. Students who reported a history of eating disorders showed an increase in eating disorder symptomatology after lockdown |
| Parola et al.  (2020) | (97) | Four Web Assessment (T1, T2, T3 & T4; 1-week intervals) in 1 month (during the lockdown) | Syndromic Scales of Adult Self-Report (ASR/18-59) | Results showed an increase in anxiety/depression, withdrawal, somatic complaints, aggressive behavior, rule-breaking behavior, and internalizing and externalizing problems from T1 to T4 |
| Pisano et al.  (2021) | (150) | Web Assessment (Self-Report Questionnaire) before (T0) and during the lockdown (T1); Web Cognitive Assessment at T1 (compared to normative data) | Depression Anxiety Stress Scale (DASS-21)  Prospective and Retrospective Memory Questionnaire (PRMQ)  Paced Auditory Serial Addition Task (PASAT)  Memory for Intentions Screening Test (MIST) | Students reported increased levels of anxiety, depression, and stress at T1 compared to T0 and an overall increase in failures in their memory skills at T1 compared to T0. Furthermore, the results showed a lower student performance at T1 for PASAT and MIST compared to normative data |
| Quarta et al. (2022) | (939) | Web Assessment (during the pandemic) | Depression Anxiety Stress Scales Short Version (DASS-21)  World Health Organization Quality of Life (WHOQoL)-Brief Questionnaire  9-item Subjective Well-Being (SWB) instrument | More than 50% of the students reported mild to extremely severe depression and stress levels, whereas 40% showed mild to extremely severe anxiety levels. Correlation analysis confirmed that physical activity and time spent in nature were associated with high mental health |
| Quintiliani et al. (2021) | (955) | Web Assessment (during the lockdown) | Perceived Stress Scale (PSS-10) Resilience Scale (RS-14) | Students reported increased stress levels in 89.4% of cases; 66% experienced moderate and 23.4% high stress. First-year students reported a greater vulnerability in their abilities to study and develop interpersonal relationships due to the COVID-19 respect to students of the other years |
| Ranieri et al.  (2021) | Younger Gen Z group (311) Older Gen Z group (218) | Web Assessment (during the pandemic) | Peritraumatic Dissociative Experiences Questionnaire (PDEQ)  COVID-19 Student Stress Questionnaire (CSSQ) Coronavirus Anxiety Scale (CAS)  Big Five Inventory-10 (BFI-10)  Affinity for e-learning Questionnaire (AEQ) | 58.9% of the students reported high-stress levels, which were higher in the younger Gen Z group. 27.1% of students reported dysfunctional anxiety. The younger Gen Z group was more vulnerable to future psychological risks |
| Renati et al.  (2023) | (399) T0 (64) T1 | Web Assessment at the beginning (during the lockdown -T0) and after a year of the pandemic (T1) | Resilience Scale for Adults (RSA)  Difficulties in Emotion Regulation Scale (DERS) Perceived Stress Scale (PSS)  Symptom Check List-90-Revised (SCL-90-R) | High levels of resilience at T0 predicted low levels of ill-being at T0 and T1. Emotional regulation skills at T0 seem to have greater weight in reducing ill-being at T0, but not at T1 |
| Romeo et al.  (2021) | (478) | Web Assessment during the lockdown | State-Trait Anxiety Inventory-Form Y1 (STAI Y1)  Beck Depression Inventory (BDI-II)  Health-related Visual Analogue Scales (VASs) | Being female, rating lower one’s health, and being more concerned about contracting COVID-19 were associated with higher anxiety symptoms. Being female, having a lower educational level (high school diploma), and rating lower one’s health were associated with higher depressive symptoms |
| Somma et al.  (2020) | (307) | Web Assessment (referring to before and during the lockdown) | Pittsburgh Sleep Quality Index (PSQI)  Beck Depression Inventory (BDI-II)  Beck Anxiety Inventory (BAI)  Personality Inventory for DSM-536-Item Form (PID-5-36) | Students reported significantly higher average PSQI scores during the lockdown than before. The Negative Affectivity dysfunctional personality domain was the leading predictor of depression and anxiety symptoms. Depression and anxiety were significant and positive predictors of poor sleep quality before and during the lockdown phase |
| Villani et al.  (2021) | (501) | Web Assessment (during the pandemic) | Zung Self-Rating Anxiety Scale (SAS)  Zung Self-Rating Depression Scale (SDS)  Patient Health Engagement Scale (PHE-S) | 35.33% of students were anxious, and 72.93% were depressed. An increase in anxiety was associated with being female, suffering from the impossibility of attending university, being distant from colleagues, and being unable to see one’s partner physically. Performing physical activity reduced this likelihood |
| Viselli et al.  (2021) | Pre-pandemic group (240) Lockdown group (240) | Web Assessment before and during the lockdown | Pittsburgh Sleep Quality Index (PSQI)  Insomnia Severity Index (ISI) Beck Depression Inventory-Second Edition (BDI-II) | The "lockdown group" reported poorer sleep quality and more severe insomnia and depression symptoms than the "pre-pandemic group." Women students were the most compromised in sleep quality, insomnia, and depressive symptoms. |
| Vitale et al.  (2020) | (285) | Web Assessment (during the lockdown) | Impact of Event Scale-Revised (IES-R) Patient Health Questionnaire, version 9 (PHQ-9) | The total value of the IES-R is significantly greater among female students than male students. Depression was more present among female students than among male students |
| Zurlo et al.  (2022) (a) | Pre-pandemic group (545) During-pandemic groups (197) (274) (200) | Web Assessment before the pandemic  Web Assessment during the pandemic | Symptom Check List-90-Revised (SCL-90-R)  COVID-19 Student Stress Questionnaire (CSSQ) Symptom Check List-90-Revised (SCL-90-R) | During the pandemic, university students reported significantly higher levels of Depression, Phobic Anxiety, Obsessions-Compulsions, and Psychoticism than the pre-pandemic group. Perceived COVID-19-related stress and psychological symptoms significantly increased as the pandemic progressed |
| Zurlo et al.  (2022) (b) | (355) | Web Assessment during the pandemic (referring to past-COVID-19 stressor and “current”- COVID-19 stressor) | COVID-19 Student Stress Questionnaire (CSSQ) Coping-Orientation-to-Problem-Experienced-New-Italian-Version (COPE-NIV)  Symptom Check List-90-Revised (SCL-90-R) | The effects of Past-COVID-19-stressors on Current- COVID-19-stressors were moderated by Coping Strategies, and the effects of Past-COVID-19-stressors on Psychological Symptoms were mediated by Current-COVID-19-stressors |

**Supplementary Material 2 – Table 2 – Summary of psychological assessment/intervention findings in university students attending counseling services.**

Table 2. Summary of psychological assessment/intervention findings in university students attending counseling services.

| **Authors (year)** | **Participants (N)** | **Type of Assessment** | **Instruments** | **Interventions** | **Main findings** |
| --- | --- | --- | --- | --- | --- |
| Celia et al.  (2022) (a) | (29) BWM-T group  (25) Control group | Web Assessment pre- (T0) and post- (T1) BWM-T intervention (during the pandemic) | Positive and Negative Affect Schedule (PANAS) State-Trait Anxiety Inventory (STAI-Y) | Four sessions of BWM-T (Brain Wave Modulation Technique) | Students in the BWM-T group reported an increase in positive affect and a decrease in negative affect after the intervention compared to the control group |
| Celia et al. (2022) (b) | (32) | Web Assessment pre- (T0) and post- (T1) intervention (during the pandemic) | Positive and Negative Affect Schedule (PANAS)  Satisfaction With Life Scale (SWLS) Young Person’s Clinical Outcomes in Routine Evaluation (YP-CORE)  State-Trait Anxiety Inventory (STAI-Y)  single-item Distress Thermometer (DT)  Zimbardo Time Perspective Inventory Short-Form (ZTPI-SF) | Five sessions of integrated mind-body approach with strategic orientation | Participants reported lower levels of negative affect, global mental distress, state and trait anxiety, and perceived stress after the intervention. Furthermore, participants reported increased positive affect, subjective well-being, and future time perspective after the intervention |
| Cerutti et al.  (2022) | (67) | Web Assessment pre- (T0) and post- (T1) intervention (during the pandemic) | Outcome Questionnaire (OQ-45)  Beck Anxiety Inventory (BAI) Beck Depression Inventory (BDI-II) Beck Hopelessness Scale (BHS)  Copenhagen Burnout Inventory (CBI) | Four sessions of psychodynamic intervention (and one 3-month follow-up session) | General functioning increased in participants between T0 and T1. Symptoms of depression, anxiety, personal burnout, and hopelessness significantly decreased between T0 and T1 |
| Cozzolino et al.  (2021) | (144) BWM-T group  (122) Control group | Web Assessment pre- (T0) and post- (T1) intervention (during the lockdown) | Single-item Distress Thermometer (DT) Perceived Stress Scale (PSS) State-Trait Anxiety Inventory (STAI-Y)  Young Person’s Clinical Outcomes in Routine Evaluation (YP-CORE)  Positive and Negative Affect Schedule (PANAS) | Four sessions of BWM-T (Brain Wave Modulation Technique) | Participants of the BWM-T group had a reduction in perceived stress, trait anxiety, state anxiety, negative affect, and global distress, and an increment in positive affect compared to participants in the control group at T1 |
| Di Consiglio et al. (2021) | (152) Before pandemic group; (74) Lockdown group;  (98) Second phase group;  (129) Third phase group | Web Assessment before the pandemic, during the lockdown, and during the pandemic | Symptom Checklist-90 Revised (SCL-90) | / | 14% of the sample met the criteria for at least one mental health disorder. During the lockdown, compared with other phases, female students reported worse symptoms in the obsessive-compulsive, interpersonal sensitivity, depression, paranoid ideation, and psychoticism dimensions |
| Gabrielli et al.  (2021) | (71) | Web Assessment pre- (T0) and post (T1) intervention (during the pandemic) | 10-item Perceived Stress Scale (PSS-10) 7-item Generalized Anxiety Disorder Scale (GAD-7)  Five-Facet Mindfulness Questionnaire (FFMQ)  User Engagement Scale – Short Form (UES-SF) | Digital mental health intervention delivered by Atena (healthy-coping chatbot intervention). Eight short sessions, each lasting about 10 minutes, delivered twice a week for 4 weeks | There was a decrease in anxiety symptoms for participants in more extreme GAD-7 score ranges and a decrease in stress symptoms as measured by the PSS-10 for all participants post-intervention |
| Giusti et al.  (2020) | (103) | Web Assessment during the lockdown | Impact of Event Scale-Revised (IES-R) 12-item General Health Questionnaire (GHQ-12) Self-Rating Anxiety Scale (SAS) Beck Depression Inventory-II (BDI-II) | / | 21.4% of students experienced lockdown as a traumatic experience. 36% of students reported anxiety symptoms, and 26% depressive symptomatology. The length of home confinement and a thinking style of “all or nothing” predicted the experience of posttraumatic symptomatology. Students having previous psychological and psychiatric contacts with mental health services (23%) showed a more severe traumatic and depressive symptomatology. |
| Ierardi et al.  (2022) | (34) pandemic group    (81) pre-pandemic group | Web Assessment pre- (T0) and post (T1) intervention    In-person Assessment pre- (T0) and post (T1) intervention | Symptom Checklist 90 Revised (SCL-90 R) Life Satisfaction Scale  Attachment Style Questionnaire (ASQ)  Adverse Childhood Experiences Questionnaire (ACE-q) | Four sessions of online psychodynamic intervention      Four sessions of in-person psychodynamic intervention | The online counseling intervention during the pandemic reduced psychological distress scales such as depression, obsessive-compulsive, interpersonal sensitivity, and anxiety.   The in-person counseling intervention effectively reduced psychological distress in all subscales and the total scale of the SCL-90 R and increased life satisfaction |
| Malighetti et al.  (2023) | (21) experimental group  (21) waiting list group (control group) | Web Assessment pre (T0) and post (T1) intervention | Perceived Stress Scale (PSS)  Mental Health Continuum-Short Form | Self-help virtual reality interventions (six sessions over a period of 3 weeks) | Students of the experimental group at T1 showed an increase in both emotional and psychological well-being compared to the control group |
| Savarese et al.  (2020) | (49) | Web Assessment during the lockdown | Symptom Checklist 90 Revised (SCL-90-R) | / | All students declared having experienced anxiety disorders, and 87% had experienced depressive symptoms. 73.5% reported sleep problems |
| Tinella et al.  (2021) | (49) | Web Assessment pre (T0) and post (T1) intervention (during the pandemic) | Psychological General Wellbeing Index (PGWBI) Emotion Regulation Questionnaire (ERQ) Perceived Self-Efficacy Scale | Five meetings with a weekly frequency and duration of 50 minutes | Increase in general psychological well-being, emotional regulation, and perceived self-efficacy scores at T1 compared to T0. Furthermore, reduction of anxiety and depression levels at T1 compared to T0 |

**Supplementary Material 3 – Table 3 – Additional information of studies included in the systematic review.**

Table 3 – Additional information of studies included in the systematic review.

| **Authors (year)** | **Participants N (F%)** | **Date(s) of data collection(s)** | **Counseling (yes/no)** | **Institution** |
| --- | --- | --- | --- | --- |
| Abenavoli et al. (2021) | 354  (68.6%) | 9 March – 4 May 2020 | No | University “Magna Grecia” of Catanzaro (only medical students) |
| Alesi et al. (2023) | 1028 (78.4%) | Merch – May 2021 | No | University of Palermo |
| Amatori et al. (2020) | 176 (47.7%) | 6 – 26 April 2020 | No | University of Urbino |
| Amerio et al. (2022) | 8177  (49.9%) | April 1 - May 1 2020 | No | University of Milan “Politecnico di Milano” |
| Baiano et al.  (2020) | 25  (40%) | November 4, 2019 –February 17, 2020 (T0)  April 26 – April 30, 2020 (T1) | No | University of Campania “Luigi Vanvitelli” |
| Baiano et al.  (2022) | 69  (71.1%) | October 2019 – February 2020 (T0)  December 2020 – February 2021 (T1) | No | University of Campania “Luigi Vanvitelli” |
| Bassi et al. (2023) | 1799 (77.5%) | April 10 – May 15 (2020) | No | University of Milano |
| Biondi et al. (2021) | 1047 (73.8%) | 1 – 7 April (2020) | No | Multicentre study |
| Bottaro & Franci (2022) | 353  (76.2%) | November 2020 – January 2021 | No | University of Enna |
| Burro et al.  (2021) | 2987  (79%) | March 23 – April 1, 2020 | No | University of Verona |
| Busetta et al. (2021) | 4379 (66%) | April 29 – May 17, 2020 | No | Multicentre study |
| Busetta et al.  (2022) | 317  (70%) | April 29 – May 17, 2020 (T0)  March 9 – April 21, 2021 (T1) | No | Multicentre study |
| Bussone  et al. (2020) | 68  (85.2%) | October 2019 (T0)  April 23 – May 4, 2020 (T1) | No | University of Rome “Sapienza” |
| Calandri et al.  (2021) | 296  (83%) | May 2020 | No | University of Torino and University of Aosta Valley |
| Calati et al.  (2022) | 1586 | April 24, 2020 – February 23, 2021 | No | University of Milano-Bicocca and University of Eastern Piedmont |
| Carletto et al.  (2022) | 1329 | December 2020 – February 2021 | No | University of Torino (only medical students) |
| Carpi et al.  (2022) | 1279 (87.4%) | March 2021 – June 2021 | No | University of Rome “Sapienza” |
| Celia et al. (2022) (a) | 54  (96.3%) | November 2020 (T0)  December 2020 (T1) | Yes | University of Foggia |
| Celia et al.  (2022) (b) | 32  (65.6%) | Late 2020 (T0)  Early 2021 (T1) | Yes | University of Foggia |
| Cellini et al. (2020) | 809 (69.3%) | March 2020 | No | University of Padova |
| Cerutti et al.  (2022) | 67 (77.6%) | May 2020 - December 2020 (T0) after four weeks (T1) | Yes | University of Roma “Sapienza” |
| Cofini et al. (2022) | 471 (71.1%) | June – July 2021 | No | University of L’Aquila |
| Commodari et al.  (2021) | 655  (72.5%) | April 22 – May 1, 2020 | No | University of Catania |
| Comparcini et al.  (2022) | 842  (76.6%) | April – July 2021 | No | Multicentre study (only nursing students) |
| Concerto et al.  (2022) | 399  (29.9%) | September 2021 – March 2022 | No | University of Catania (only medical students) |
| Conti et al.  (2023) | 177  (89.3%) | October – December 2019 (T0)  March – May 2021 (T1) | No | University of Chieti |
| Cozzolino et al.  (2021) | 266  (77.7%) | March 2020 (T0)  April 2020 (T1) | Yes | University of Salerno |
| De Pasquale et al.  (2021) (a) | 194  (55.6%) | September 2020 – January 2021 | No | University of Catania |
| De Pasquale et al.  (2021) (b) | 469 (52.8%) | March 2020 – February 2021 | No | University of Catania |
| Di Consiglio et al. (2021) | 454 (84.2%) | March 2019 (T0) March 2020 – May 2020 (T1) October 2020 – December 2020 (T2)  January 2021 – March 2021 (T3) | Yes | University of Roma “Sapienza” |
| Fornili et al.  (2021) | 14028 | April – May 2020 | No | Multicentre study |
| Gabrielli et al.  (2021) | 71 (68%) | October 2020 (T0)  November 2020 (T1) | Yes | University of Trento |
| Generali et al.  (2020) | 399 (56.9%) | April 21 – May 5, 2020 | No | Multicentre study (only dental students) |
| Giangrasso et al.  (2022) | 350 (79.7%) | April 2020 | No | University of Florence |
| Giusti et al.  (2020) | 103  (81.6%) | March 16 – May 4, 2020 | Yes | University of L’Aquila |
| Giusti et al.  (2021) | 203  (76.4%) | July 15 – September 30, 2020 | No | University of L’Aquila |
| Guidotti et al.  (2022) | 78  120  (81.6%) | 2019 (pre-pandemic group)  April – July 2022 (pandemic-group) | No | University of Parma |
| Ieradi et al.  (2022) | 34  (79%)  81 (74%) | January 2020 (T0) - July 2021 (T1) (pandemic group)  January 2016 (T0) – December 2019 (T1) (pre-pandemic group) | Yes | University of Milano-Bicocca |
| Lo Moro et al.  (2022) | 121 (longitudinal sub-sample)  (68.6%)  705 (67.2%) 283 (pre-pandemic group) 422 (pandemic group) | November 2018 (T0)  December 2020 – February 2021 (T1)  November 2018 December 2020 – February 2021 | No | University of Turin (only medical students) |
| Loscalzo & Giannini  (2021) | 6075 (74.6%) | March – June 2020 | No | University of Florence |
| Malighetti et al.  (2023) | 42 (76.2%) |  | Yes | Università Cattolica del Sacro Cuore (Milan) |
| Manfredi et al., (2022) | 90  (43.3%) | November 2021 – March 2022 | No | University of Brescia (only medical students) |
| Marelli et al.  (2021) | 307  (74.9%) | March 24 – May 3, 2020 | No | University Vita-Salute San Raffaele (Milan) |
| Marzilli et al. (2022) | 410 (71.2%) | 15 November 2020 – 15 March 2021 | No | University of Roma “Sapienza” |
| Meda et al.  (2021) | 161  197 | October 2019 (T0) – April 2020 (T1)  November, December 2019 (T0) – May, June 2020 (T1) | No | University of Padova |
| Parola et al.  (2020) | 97  (49.5%) | March – April 2022 - four assessment (T1, T2, T3 & T4; 1-week intervals) in 1 month | No | University of Naples “Federico II” |
| Pisano et al.  (2021) | 150  (100%) | February (T0) – April 2020 (T1) | No | University of Naples “Federico II” |
| Quarta et al.  (2022) | 939 (75%) | April – May 2021 | No | University of Salento |
| Quintiliani et al. (2021) | 955 (68.5%) | April, 10 – May, 3 2020 | No | Campus Bio Medico University of Rome |
| Ranieri et al.  (2021) | 529 (79.6%) | December 2020 | No | University of L’Aquila |
| Renati et al.  (2023) | 399 (T0) 64 (T1) | April 2020 (T0) May 2021 (T1) | No | University of Cagliari |
| Romeo et al.  (2021) | 478 (77.4%) | March 19 – April 5, 2020 | No | University of Turin |
| Savarese et al.  (2020) | 266 | March 12 – May 3, 2020 | Yes | University of Salerno |
| Somma et al.  (2020) | 307  (74.9%) | March 2020 | No | Vita-Salute San Raffaele University of Milan |
| Tinella et al.  (2021) | 49 (75.5%) | April 16 – October 10, 2020 (T0) after five weeks (T1) | Yes | University of Bari “Aldo Moro” |
| Villani et al.  (2021) | 501 (71.4%) | June 8 – July 12, 2020 | No | Università Cattolica del Sacro Cuore (Milan, Brescia, Piacenza-Cremona, Rome) |
| Viselli et al.  (2021) | 240 (pre-pandemic group) 240 (lockdown group) (80.4%) | October 6 – October 11, 2016 March 25 – March 31, 2020 | No | University of L’Aquila |
| Vitale et al.  (2020) | 285 (85.9%) | March 25 – April 25, 2020 | No | Multicentre Study (only nursing student) |
| Zurlo et al.  (2022) (a) | 545 (Pre-pandemic group)  (75.4%)  671 (During-pandemic groups) (75%), 197, 274, 200 | 2017 (T0)   April 2020  November 2020  April 2021 | No | University of Naples “Federico II” |
| Zurlo et al.  (2022) (b) | 355 (63.4%) | April – June 2022 | No | University of Naples “Federico II” |

**Supplementary Material 4 – Table 4 and 5, and Figure 4. Risk of bias scores in each study.**

Table 4 – Risk of bias of cross-sectional studies.

| **Cross-sectional studies (n=43)** | **Selection** | | | | **Comparability** | **Outcome** | | **Total**  **(0-10) *** |
| --- | --- | --- | --- | --- | --- | --- | --- | --- |
|  | *Representativeness of the sample* | *Sample size* | *Non-respondents* | *Ascertainment of the exposure* | *Adjustment of the outcome* | *Assessment of the outcome* | *Statistical test* |  |
| Abenavoli et al. (2021) | 1 | 1 | 0 | 2 | 1 | 1 | 1 | 7 |
| Alesi et al. (2023) | 1 | 1 | 0 | 2 | 2 | 1 | 1 | 8 |
| Amatori et al. (2020) | 1 | 1 | 1 | 2 | 2 | 1 | 1 | 9 |
| Amerio et al. (2022) | 1 | 1 | 1 | 2 | 2 | 1 | 1 | 9 |
| Bassi et al. (2023) | 1 | 1 | 1 | 2 | 2 | 1 | 1 | 9 |
| Biondi et al. (2021) | 1 | 1 | 0 | 2 | 2 | 1 | 1 | 8 |
| Bottaro & Franci (2022) | 1 | 0 | 0 | 2 | 1 | 1 | 1 | 6 |
| Burro et al. (2021) | 1 | 1 | 0 | 2 | 2 | 1 | 1 | 8 |
| Busetta et al. (2021) | 1 | 1 | 1 | 2 | 2 | 1 | 1 | 9 |
| Calandri et al. (2021) | 1 | 1 | 1 | 2 | 2 | 1 | 1 | 9 |
| Calati et al. (2022) | 1 | 1 | 1 | 2 | 2 | 1 | 1 | 9 |
| Carletto et al. (2022) | 1 | 1 | 1 | 2 | 2 | 1 | 1 | 9 |
| Carpi et al. (2022) | 1 | 1 | 1 | 2 | 2 | 1 | 1 | 9 |
| Cellini et al. (2020) | 1 | 1 | 1 | 2 | 1 | 1 | 1 | 8 |
| Cofini et al. (2022) | 1 | 1 | 0 | 2 | 2 | 1 | 1 | 8 |
| Commodari et al. (2021) | 1 | 1 | 0 | 2 | 2 | 1 | 1 | 8 |
| Comparcini et al. (2022) | 1 | 1 | 0 | 2 | 2 | 1 | 1 | 8 |
| Concerto et al. (2022) | 1 | 1 | 0 | 2 | 1 | 1 | 1 | 7 |
| De Pasquale et al. (2021) (a) | 1 | 0 | 0 | 2 | 1 | 1 | 1 | 6 |
| De Pasquale et al. (2021) (b) | 1 | 1 | 0 | 2 | 2 | 1 | 1 | 8 |
| Di Consiglio et al. (2021) | 1 | 1 | 1 | 2 | 2 | 1 | 1 | 9 |
| Fornili et al. (2021) | 1 | 1 | 1 | 2 | 2 | 1 | 1 | 9 |
| Generali et al. (2020) | 1 | 1 | 1 | 2 | 2 | 1 | 1 | 9 |
| Giangrasso et al. (2022) | 1 | 1 | 0 | 2 | 2 | 1 | 1 | 8 |
| Giusti et al. (2020) | 1 | 1 | 0 | 2 | 2 | 1 | 1 | 8 |
| Giusti et al. (2021) | 1 | 1 | 0 | 2 | 2 | 1 | 1 | 8 |
| Guidotti et al. (2022) | 1 | 1 | 0 | 2 | 2 | 1 | 1 | 8 |
| Lo Moro et al. (2022) | 1 | 1 | 0 | 2 | 2 | 1 | 1 | 8 |
| Loscalzo & Giannini (2021) | 1 | 1 | 0 | 2 | 2 | 1 | 1 | 8 |
| Manfredi et al. (2021) | 1 | 1 | 0 | 2 | 2 | 1 | 1 | 8 |
| Marelli et al. (2021) | 1 | 1 | 0 | 2 | 2 | 1 | 1 | 8 |
| Marzilli et al. (2022) | 1 | 1 | 1 | 2 | 2 | 1 | 1 | 9 |
| Quarta et al. (2022) | 1 | 1 | 1 | 2 | 2 | 1 | 1 | 9 |
| Quintiliani et al. (2021) | 1 | 1 | 0 | 2 | 1 | 1 | 1 | 7 |
| Ranieri et al. (2021) | 1 | 1 | 0 | 2 | 1 | 1 | 1 | 7 |
| Romeo et al. (2021) | 1 | 1 | 0 | 2 | 2 | 1 | 1 | 8 |
| Savarese et al. (2020) | 1 | 1 | 1 | 2 | 1 | 1 | 0 | 7 |
| Somma et al. (2020) | 1 | 1 | 1 | 2 | 2 | 1 | 1 | 9 |
| Villani et al. (2021) | 1 | 1 | 1 | 2 | 2 | 1 | 1 | 9 |
| Viselli et al. (2021) | 1 | 1 | 0 | 2 | 2 | 1 | 1 | 8 |
| Vitale et al. (2020) | 1 | 1 | 0 | 2 | 1 | 1 | 1 | 7 |
| Zurlo et al. (2022) (a) | 1 | 1 | 0 | 2 | 2 | 1 | 1 | 8 |
| Zurlo et al. (2022) (b) | 1 | 1 | 0 | 2 | 2 | 1 | 1 | 8 |

Table 5 – Risk of bias of cohort studies.

| **Cohort studies (n=15)** | **Selection** | | | | **Comparability** | **Outcome** | | | **Total**  **(0-9) *** |
| --- | --- | --- | --- | --- | --- | --- | --- | --- | --- |
|  | *Representativeness of the exposed sample* | *Selection of the non-exposed cohort* | *Ascertainment of exposure* | *Demonstration that outcome of interest was not present at start of study* | *Comparability of cohorts on the basis of the design or analysis controlled for confounders* | *Assessment of outcome* | *Was follow-up long enough for outcomes to occur?* | *Adequacy of follow-up of cohorts* |  |
| Baiano et al. (2020) | 1 | 0 | 0 | 1 | 1 | 0 | 1 | 1 | 5 |
| Baiano et al. (2022) | 1 | 0 | 0 | 1 | 1 | 0 | 1 | 1 | 5 |
| Busetta et al. (2022) | 1 | 0 | 0 | 1 | 1 | 0 | 1 | 1 | 5 |
| Bussone et al. (2020) | 1 | 0 | 0 | 1 | 1 | 0 | 1 | 1 | 5 |
| Celia et al. (2022) (b) | 1 | 0 | 0 | 1 | 1 | 0 | 1 | 1 | 5 |
| Cerutti et al. (2022) | 1 | 0 | 0 | 1 | 1 | 0 | 1 | 1 | 5 |
| Conti et al. (2023) | 1 | 0 | 0 | 1 | 1 | 0 | 1 | 1 | 5 |
| Lo Moro et al. (2022) | 1 | 0 | 0 | 1 | 1 | 0 | 1 | 1 | 5 |
| Gabrielli et al. (2021) | 1 | 0 | 0 | 1 | 1 | 0 | 1 | 1 | 5 |
| Ierardi et al. (2022) | 1 | 1 | 0 | 1 | 1 | 0 | 1 | 1 | 6 |
| Meda et al. (2021) | 1 | 1 | 0 | 1 | 1 | 0 | 1 | 1 | 6 |
| Parola et al. (2020) | 1 | 0 | 0 | 1 | 1 | 0 | 1 | 1 | 5 |
| Pisano et al. (2021) | 1 | 0 | 1 | 1 | 1 | 0 | 1 | 1 | 6 |
| Renati et al. (2023) | 1 | 0 | 0 | 1 | 1 | 0 | 1 | 1 | 5 |
| Tinella et al. (2021) | 1 | 0 | 0 | 1 | 1 | 0 | 1 | 1 | 5 |

Figure 4 – Risk of bias of randomized trials.


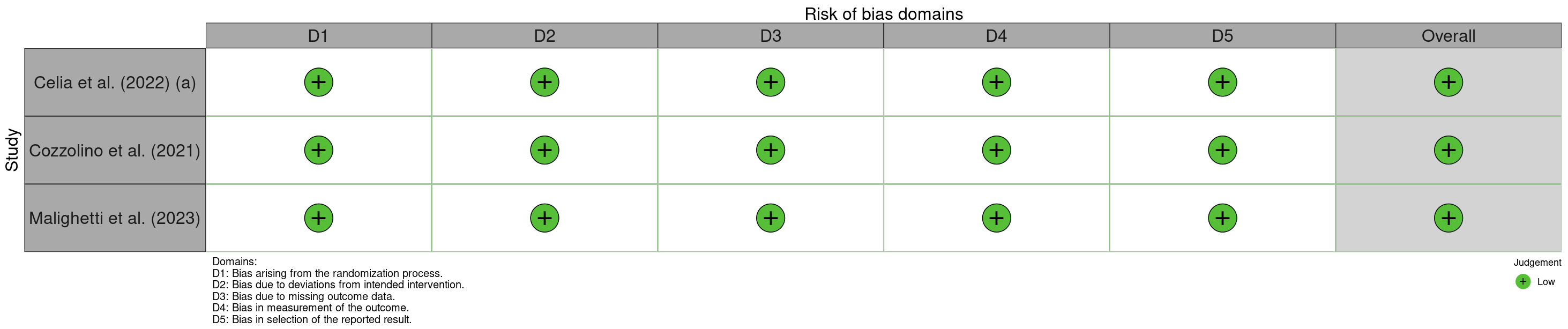


**Supplementary Material 5 – Literature search and results**

PUBMED

("COVID" OR "CORONA" OR "SARS-COV-2") AND (ITAL*) AND ("SCHOOL*" OR "UNIVERSIT*" OR "COLLEGE*" OR "HIGHER EDUCATION" OR "ACADEMIC" OR "INSTITUTION OF HIGHER LEARNING" OR "MASTER*") AND (“ASSESSMENT*" OR “EVALUATION*" OR “VALUATION*” OR “SCREEN*” OR “TEST” OR “INTERVENTION*” OR “THERAP*” OR “TREATMENT*”) AND (“PSYCH*" OR “COGNIT*” OR "COUNSELING" OR "PSYCHOLOGICAL COUNSELING" OR "PSYCHOLOGICAL CONSULTATION") AND ("DEPRESS*" OR "ANXIETY" OR "EMOT*" OR "STRESS" OR "DISTRESS" OR "PANIC" OR "PSYCHOLOGICAL DISCOMFORT*" OR "PSYCHOLOGICAL DISEASE*" OR "PSYCHOLOGICAL DISORDER*" OR "EMOTIONAL DISEASE*" OR “EMOTIONAL DISCOMFORT*" OR "EMOTIONAL DISORDER*" OR "MENTAL DISEASE*" OR "MENTAL DISCOMFORT*" OR "MENTAL DISORDER*" OR "MENTAL HEALTH CONDITION*”)

Date of search: April 14, 2023; Filter applied: Years: 2019-2023; Results: 1361

WEB OF SCIENCE

ALL=("COVID" OR "CORONA" OR "SARS-COV-2") AND ALL=(ITAL*) AND ALL=("SCHOOL*" OR "UNIVERSIT*" OR "COLLEGE*" OR "HIGHER EDUCATION" OR "ACADEMIC" OR "INSTITUTION OF HIGHER LEARNING" OR "MASTER*") AND ALL=(“ASSESSMENT*" OR “EVALUATION*" OR “VALUATION*” OR “SCREEN*” OR “TEST” OR “INTERVENTION*” OR “THERAP*” OR “TREATMENT*”) AND ALL=(“PSYCH*" OR “COGNIT*” OR "COUNSELING" OR "PSYCHOLOGICAL COUNSELING" OR "PSYCHOLOGICAL CONSULTATION") AND ALL=("DEPRESS*" OR "ANXIETY" OR "EMOT*" OR "STRESS" OR "DISTRESS" OR "PANIC" OR "PSYCHOLOGICAL DISCOMFORT*" OR "PSYCHOLOGICAL DISEASE*" OR "PSYCHOLOGICAL DISORDER*" OR "EMOTIONAL DISEASE*" OR “EMOTIONAL DISCOMFORT*" OR "EMOTIONAL DISORDER*" OR "MENTAL DISEASE*" OR "MENTAL DISCOMFORT*" OR "MENTAL DISORDER*" OR "MENTAL HEALTH CONDITION*”)

Date of search: April 14, 2023; Filters applied: Pubblication Years: 2019-2023; Languages: English, Italian; Countries/Regions: Italy; Document Types: Article; Results: 954

SCOPUS

TITLE-ABS-KEY("COVID" OR "CORONA" OR "SARS-COV-2") AND TITLE-ABS-KEY(ITAL*) AND TITLE-ABS-KEY("SCHOOL*" OR "UNIVERSIT*" OR "COLLEGE*" OR "HIGHER EDUCATION" OR "ACADEMIC" OR "INSTITUTION OF HIGHER LEARNING" OR "MASTER*") AND TITLE-ABS-KEY(“ASSESSMENT*" OR “EVALUATION*" OR “VALUATION*” OR “SCREEN*” OR “TEST” OR “INTERVENTION*” OR “THERAP*” OR “TREATMENT*”) AND TITLE-ABS-KEY(“PSYCH*" OR “COGNIT*” OR "COUNSELING" OR "PSYCHOLOGICAL COUNSELING" OR "PSYCHOLOGICAL CONSULTATION") AND TITLE-ABS-KEY("DEPRESS*" OR "ANXIETY" OR "EMOT*" OR "STRESS" OR "DISTRESS" OR "PANIC" OR "PSYCHOLOGICAL DISCOMFORT*" OR "PSYCHOLOGICAL DISEASE*" OR "PSYCHOLOGICAL DISORDER*" OR "EMOTIONAL DISEASE*" OR “EMOTIONAL DISCOMFORT*" OR "EMOTIONAL DISORDER*" OR "MENTAL DISEASE*" OR "MENTAL DISCOMFORT*" OR "MENTAL DISORDER*" OR "MENTAL HEALTH CONDITION*”)

Date of search: April 14, 2023; Filters applied: Language: English, Italian; Country/Territory: Italy; Document Type: Article; Results: 155

EBSCOhost (APA PsycInfo)

("COVID" OR "CORONA" OR "SARS-COV-2") AND (ITAL*) AND ("SCHOOL*" OR "UNIVERSIT*" OR "COLLEGE*" OR "HIGHER EDUCATION" OR "ACADEMIC" OR "INSTITUTION OF HIGHER LEARNING" OR "MASTER*") AND (“ASSESSMENT*" OR “EVALUATION*" OR “VALUATION*” OR “SCREEN*” OR “TEST” OR “INTERVENTION*” OR “THERAP*” OR “TREATMENT*”) AND (“PSYCH*" OR “COGNIT*” OR "COUNSELING" OR "PSYCHOLOGICAL COUNSELING" OR "PSYCHOLOGICAL CONSULTATION") AND ("DEPRESS*" OR "ANXIETY" OR "EMOT*" OR "STRESS" OR "DISTRESS" OR "PANIC" OR "PSYCHOLOGICAL DISCOMFORT*" OR "PSYCHOLOGICAL DISEASE*" OR "PSYCHOLOGICAL DISORDER*" OR "EMOTIONAL DISEASE*" OR “EMOTIONAL DISCOMFORT*" OR "EMOTIONAL DISORDER*" OR "MENTAL DISEASE*" OR "MENTAL DISCOMFORT*" OR "MENTAL DISORDER*" OR "MENTAL HEALTH CONDITION*”)

Date of search: April 14, 2023; Filters applied: Years: 2019-2023; Source Types: Academic Journals; Results: 419
